# Isothermal Experiments on Steam Oxidation of Zr−Sn−Nb Alloy at 1050 °C: Kinetics and Process

**DOI:** 10.3390/ma16103823

**Published:** 2023-05-18

**Authors:** Rui Jiang, Dewen Tang, Chen Yang, Yanli Wang, Lin Zhang, Ming Lei

**Affiliations:** 1 School of Mechanical Engineering, University of South China, Hengyang 421001, China; jr5201314ct@163.com (R.J.); yangchen2023@163.com (C.Y.); endeavor09@163.com (Y.W.); 2 Hunan Provincial Key Laboratory of Emergency Safety Technology and Equipment for Nuclear Facilities, University of South China, Hengyang 421001, China; 3 Nuclear Power Institute of China, Chengdu 610213, China; 4 China National Nuclear Corporation Jianzhong Nuclear Fuel Company Ltd., Yibin 644603, China

**Keywords:** Zr−Sn−Nb alloy, steam oxidation, oxidation kinetics, oxidation characteristics

## Abstract

The isothermal steam oxidation behavior of the Zr−Sn−Nb alloy at 1050 °C was studied. In this study, the oxidation weight gain of Zr−Sn−Nb samples with oxidation durations ranging from 100 s to 5000 s was calculated. The oxidation kinetic properties of the Zr−Sn−Nb alloy were obtained. The macroscopic morphology of the alloy was directly observed and compared. The microscopic surface morphology, cross-section morphology, and element content of the Zr−Sn−Nb alloy were analyzed using scanning electron microscopy (SEM), transmission electron microscopy (TEM), X-ray diffraction (XRD), and energy disperse spectroscopy (EDS). According to the results, the cross-sectional structure of the Zr−Sn−Nb alloy consisted of ZrO_2_, α-Zr(O), and prior-β. During the oxidation process, its weight gain versus oxidation time curve followed a parabolic law. The thickness of the oxide layer increases. Micropores and cracks gradually appear on the oxide film. Similarly, the thicknesses of ZrO_2_ and α-Zr versus oxidation time were in accordance with the parabolic law.

## 1. Introduction

Zirconium alloys are used as cladding materials for nuclear reactor fuel elements due to their good corrosion resistance, low thermal neutron absorption rate, and excellent mechanical properties [1,2]. However, it has been reported that zirconium alloys are disadvantageous in high-temperature water vapor because the oxide film produced by the oxidation reaction of metal zirconium adheres to the surface, which affects the performance of the alloy [3,4]. As the temperature of the core rises during a loss-of-coolant accident (LOCA), the cladding reacts violently with the high-temperature water vapor, resulting in the α to β transformation of the zirconium alloy matrix [5,6,7]. Moreover, brittle phases including ZrO_2_ and α-Zr(O) are formed. Zirconium alloys are easily damaged due to the pressure inside the cladding tubes. Moreover, in the Fukushima accident in Japan in 2011 [8,9], oxidation of zirconium metal and water/steam occurred, releasing a large amount of hydrogen gas [10]. This led to an explosion which posed a hazard to human society and the natural environment. Therefore, it is of great significance to study the high-temperature steam oxidation behavior of zirconium alloys in order to ensure the safe operation of nuclear reactors.

To ensure the safety and reliability of the fuel assemblies, the Nuclear Regulatory Commission (NRC) has established a requirement that a maximum cladding temperature of 1204 °C and an equivalent cladding reacted (ECR) of 17% should not be exceeded under LOCA conditions. In addition, the oxidation kinetics of zirconium alloys such as Zr-2 and Zr-4 have been studied [11,12,13,14,15,16,17]. Steinbruck et al. [18] reported that the oxidation kinetics of the E110 alloy are similar to those of the Zr-4 alloy, but the oxidation rate of the former is lower, above the temperature of 1100 °C. Jong et al. [19] believed that the kinetic curve follows a parabolic law in the range of 800 °C to 1050 °C after studying the water vapor oxidation kinetics of the Zr-1 Nb-1 Sn-0.1 Fe alloy. In contrast, the changes in oxidation kinetics are mainly related to the phase transformation of the zirconium alloy matrix and oxidation products. Baker [20] and Cathcart [21] et al. obtained a steam oxidation reaction expression of a zirconium alloy, and it is considered that the high-temperature steam oxidation reaction obeys the parabolic law.

Furthermore, it has been reported that the amount of oxidative corrosion of zirconium alloys increases with the increasing fuel cycle period. Therefore, it is necessary to study the performance improvement of zirconium alloys and to develop more advanced zirconium alloys. In recent years, various types of zirconium alloys have been developed and used in engineering applications, including ZIRLO alloys, M5 alloys, E635 alloys, etc. Among them, HA-NA alloys, N18 alloys, and NDA alloys, etc., have good application prospects [22,23,24,25,26]. However, studies on the oxidation mechanism of zirconium alloys in high-temperature steam are still limited. Moreover, the oxidation behavior of zirconium alloys can be influenced by different oxidation temperatures or times. To date, mechanistic studies of the effect of Sn on the high-temperature oxidation properties of zirconium alloys have been overwhelmingly conducted on more conventional Zr alloys that do not contain any Nb. Currently, Nb-containing alloys such as ZIRLO and M5 have widely replaced conventional zirconium alloys in PWR fuel assemblies, while composition design based on the Wagner–Hauffe hypothesis guiding the oxidation corrosion resistance of zirconium alloys suggests that alloying with zirconium cohort elements is theoretically the most beneficial for improving the corrosion resistance of zirconium [27]. Therefore, we investigated a new zirconium alloy containing Nb-1.0, Sn-1.0, Fe-0.3, and O-0.1 and performed the relevant experiments on it.

In this paper, the isothermal oxidation behavior of Zr−Sn−Nb alloys under a steam environment at 1050 °C was investigated. The weight gain results were presented for Zr−Sn−Nb alloys that were oxidized at 100 s, 500 s, 1000 s, 2500 s, 4000 s, and 5000 s under high-temperature steam conditions. Then, to reveal the pattern of their steam oxidation, the weight gains and thicknesses of ZrO_2_ and α-Zr were analyzed in detail. In addition, macroscopic characteristics were compared by direct observation. The microscopic surface, cross-section morphology, and cross-section distribution of the elements were observed using electron microscopy (SEM), transmission electron microscopy (TEM), X-ray diffraction (XRD), and energy disperse spectroscopy (EDS). These processes should be performed to fully understand the oxidation kinetics and mechanisms of the Zr−Sn−Nb alloy.

## 2. Materials and Methods

### 2.1. Sample Preparation

**Figure 1:**
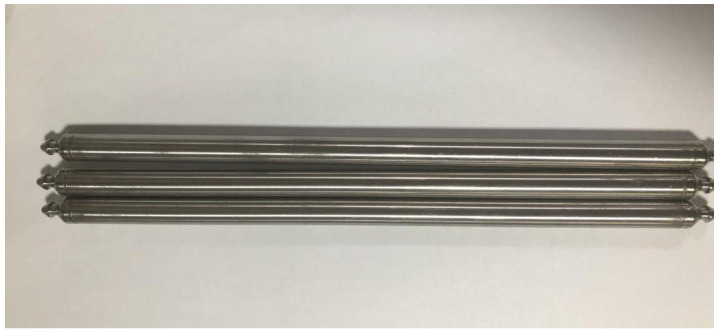
Zr−Sn−Nb alloy single-sided tube samples.

As shown in Figure 1, the experimental material was an annealed Zr−Sn−Nb alloy tube with a length of 216.0 mm, an outer diameter of 9.20 mm, and a thickness of 1.00 mm. The main elements in the Zr−Sn−Nb alloy were Nb-1.0, Fe-0.3, O-0.1, Sn-1.0, and Zr-balance. After oxidation, the sample was welded with end plugs at both ends of the cladding tube by means of ring welding, with the smallest possible end plug size. Prior to end plug welding, the sample was evacuated or filled with steam to ensure that the internal pressure of the sample was lower than the steam pressure at the oxidation temperature. In addition, the samples used in the experiment conformed to the technical standards for the processing and manufacturing of cladding tubes for nuclear power.

### 2.2. Experimental Method

The oxidized samples were put into the NBD-LT1700 high-temperature steam oxidation furnace; oxidized at 1050 °C for 100 s, 500 s, 1000 s, 2500 s, 4000 s, and 5000 s; and then air-cooled to room temperature. During the experiment, excessive heating and cooling time were avoided (the total duration of heating and cooling should not exceed 10% of the duration of the entire oxidation experiment). The heating rate was not intended to be less than 5 °C/s. In addition, the water quality was grade A, as specified by ASTM G2/G2M. The average steam flow was between (0.5~30) mg/(cm^2^·s). The vapor pressure was generally 0.1 MPa.

Before and after oxidation by high-temperature steam, the mass of the sample was measured by an electronic analytical balance with an accuracy of 0.1 mg. Equation (1) was used to calculate the oxidation weight gain W of the sample:

(1) $$W=\frac{\left(W_{t}-W_{0}\right)\left(\mathrm{mg}\right)}{A\left({\mathrm{cm}}^{2}\right)}$$

where W_0_ is the initial mass of the oxidized sample, $W_{t}$ is the mass of the sample corresponding to the oxidation duration, and A is the surface area of the sample that was exposed to steam.

The samples were made into metallographic samples after high-temperature steam oxidation. After grinding and polishing the cross-sections of the samples, the cross-sections and surface micromorphology of the alloys were analyzed by scanning electron microscopy (SEM) and transmission electron microscopy (TEM). Then, the content of elements in the cross-sectional structure of Zr−Sn−Nb alloy was analyzed by an energy dispersive spectrometer (EDS). The thicknesses of the oxygen-saturated ZrO_2_ and α-Zr(O) layers in SEM images were measured using Image Pro Plus software, and the oxygen content distribution in each phase layer was measured. Finally, all of the obtained data were fitted into curves using Origin software.

## 3. Results

### 3.1. Macroscopic Appearance

**Figure 2:**
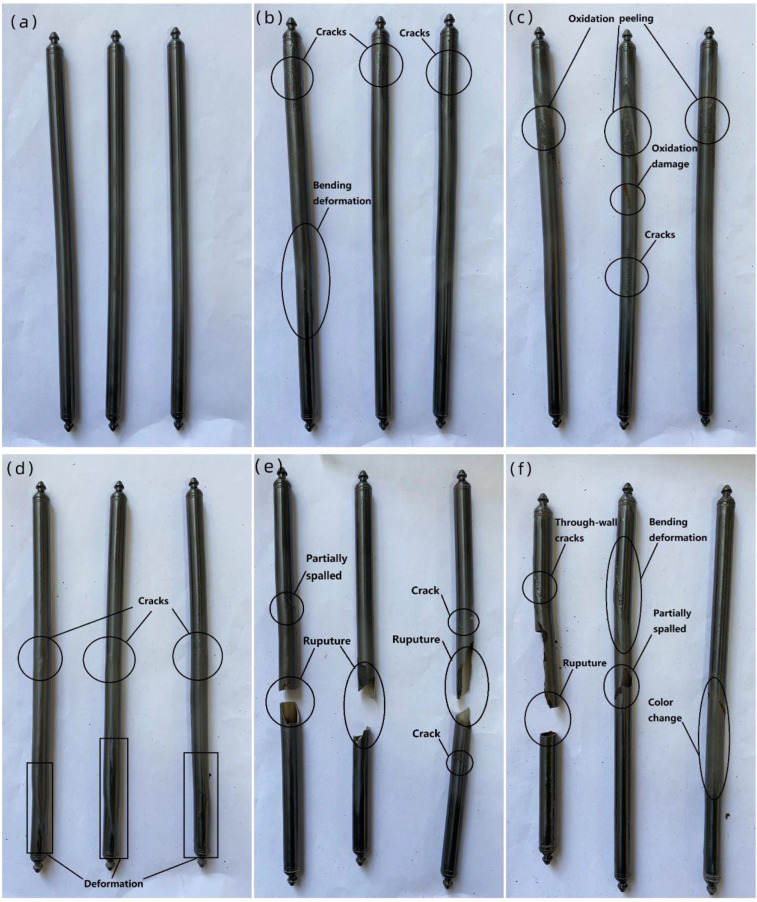
Macroscopic appearance of Zr−Sn−Nb alloy after steam oxidation at 1050 °C for (**a**) 100 s, (**b**) 500 s, (**c**) 1000 s, (**d**) 2500 s, (**e**) 4000 s, and (**f**) 5000 s.

Figure 2 shows the macroscopic appearance of the Zr−Sn−Nb alloy after steam oxidation at 1050 °C for 100 s, 500 s, 1000 s, 2500 s, 4000 s, and 5000 s (Figure 2a–f). It can be seen that as oxidation proceeded, the structure of the Zr−Sn−Nb alloy was overall bent or locally extended, and the skin gradually peeled off. The oxidation phenomena appearing in the macroscopic morphology can be better explained by observing the microscopic surface morphology of the Zr−Sn−Nb alloy. At 500 s of oxidation, the pores on the surface structure of the sample increased significantly, which explains the darker color in Figure 2b due to the absorption of visible light by the micropores. As the oxidation time proceeded on, the transverse and longitudinal cracks on the microscopic surface increased, the crack width became larger, the pores expanded and cracked, and even the spalling phenomenon became obvious. They broke from the middle (Figure 2e−f). Compared with the macroscopic structural changes at oxidation times of 100 s, 500 s, and 1000 s (Figure 2a–c), the structural changes at oxidation times of 2500 s, 4000 s, and 5000 s (Figure 2d–f) were more drastic. The samples cracked after 4000 s of oxidation (Figure 2e−f).

### 3.2. Oxidation Weight Gain

**Figure 3:**
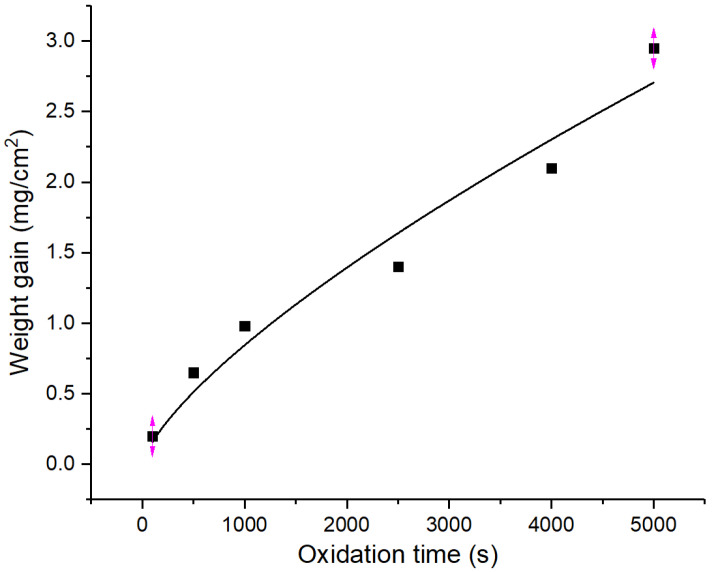
Variation curve of Zr−Sn−Nb alloy’s weight gain by steam oxidation at 1050 °C.

The kinetic curve of oxidation weight gain of the Zr−Sn−Nb alloy at 1050 °C is shown in Figure 3, and the data of time versus weight gain are shown in Table 1. From Figure 3, it can be seen that the kinetic oxidation curve conformed to the parabolic law of $W=at^{b}$ when the oxidation time was in the range of 0~5000 s. The fitted functional expressions are shown in Table 2. Figure 3 also shows that the weight gain per unit area of the Zr−Sn−Nb alloy generally increased with the oxidation time, but the weight gain values were different at different times, indicating that the oxidation time was one of the factors affecting the oxidation rate.

In the early stage of oxidation, most of the oxide film is black, with a relatively dense distribution and the presence of small pits, but no obvious bulging and cracking. At this time, the change in the oxidation weight gain is determined by the diffusion rate of oxygen in the oxide layer, which is in accordance with the parabolic law. In addition, the dense oxide film can hinder the oxygen’s diffusion into the alloy substrate surface so that the oxidation rate is kept at a low level. However, as the oxidation corrosion deepens, more and more Zr−Sn−Nb alloy matrix transitions from the α to the β phase, and the transition rate also accelerates, which promotes the transformation of the mechanical properties of the alloy matrix, the internal stress of the oxide film, the bonding state of the oxide film, and the matrix change, resulting in the oxide film turning gray and bulging, as well as the formation of small holes and cracks. With the expansion of the size of the pores and cracks, and even the peeling of the skin, the oxygen diffusion is accelerated and the oxidation weight gain in the later stages of oxidation gradually changes from a parabolic law to a linear law.

Yanzhang Liu [28] et al. studied the oxidation kinetics of the N18 alloy under high-temperature steam oxidation. They concluded that the kinetic curve of weight gain versus oxidation time gradually changed from a parabolic law to a cubic exponential law as the oxidation temperature increased. The oxidation data of the Zr−Sn−Nb alloy at 1050 °C were fitted by the least squares method during a steam oxidation time of less than 2500 s, and the weight gain versus time was obtained in accordance with the parabolic law, as shown in Equation (2), which is very close to the research results of Cathcat [21] and Leistikow [29].

(2) Wt=Kt12

where $W_{t}$ is the weight gain when the oxidation time is *t*, K is the oxidation rate constant, and *t* is the oxidation time.

By substituting the different oxidation times and oxidative weight gain values in Table 1 into Equation (2), and by plotting $W_{t}$ against t12, the value of slope K can be obtained as 0.04091.

### 3.3. Surface Morphology

**Figure 4:**
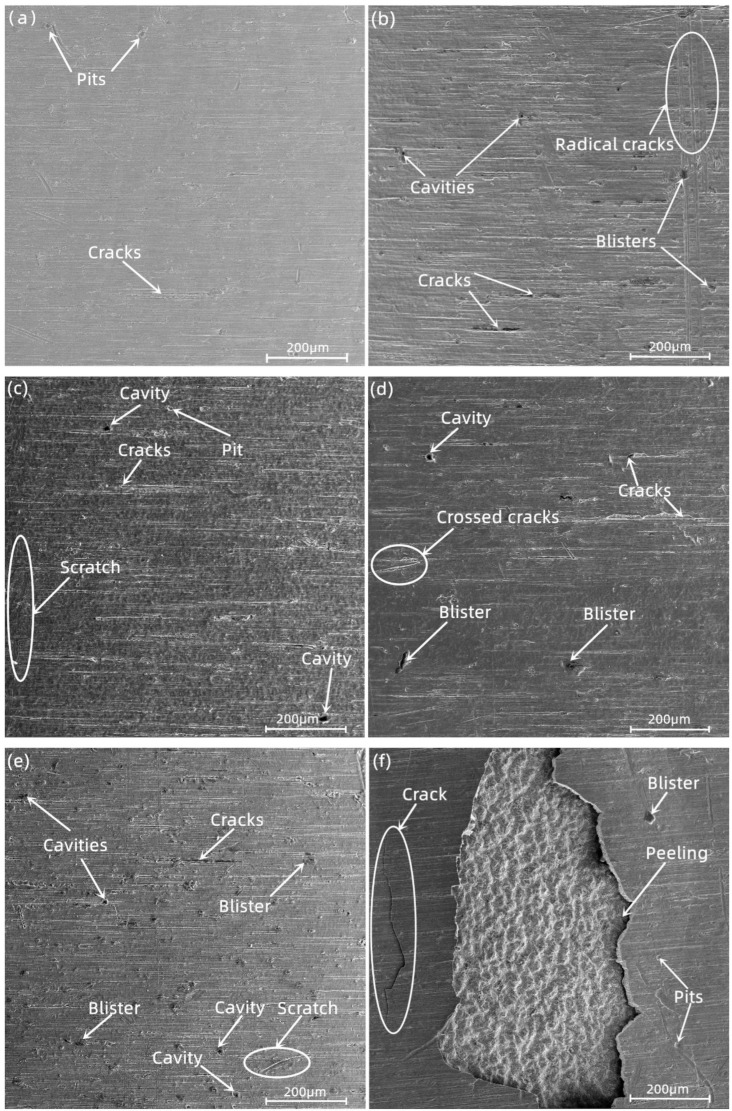
Surface morphology of Zr−Sn−Nb alloy after steam oxidation at 1050 °C for (**a**) 100 s, (**b**) 500 s, (**c**) 1000 s, (**d**) 2500 s, (**e**) 4000 s, and (**f**) 5000 s.

Figure 4 shows the surface microstructure of the Zr−Sn−Nb alloy when subjected to different durations of steam oxidation at 1050 °C. It can be observed that with the increase in oxidation time, the number of cracks on the surface of the alloy increased, the width of the cracks became larger, and holes of different sizes gradually appeared. After 100 s of oxidation, the surface was relatively smooth, but there were also tiny cracks and irregularly distributed pits. At oxidation times of 500 s, 1000 s, and 2500 s, the cracks became more obvious, and were accompanied by the proliferation of holes with localized areas of cracking. When the oxidation time ranged from 4000 s to 5000 s, a large number of holes appeared in the area surrounded by cracks, plate cracks appeared, and there was obvious spalling on the surface (Figure 4f). The diffusion area of oxygen elements expanded due to the increase in the number of cracks, thus accelerating the oxidation of the alloy’s surface.

### 3.4. Cross-Sectional Morphology

**Figure 5:**
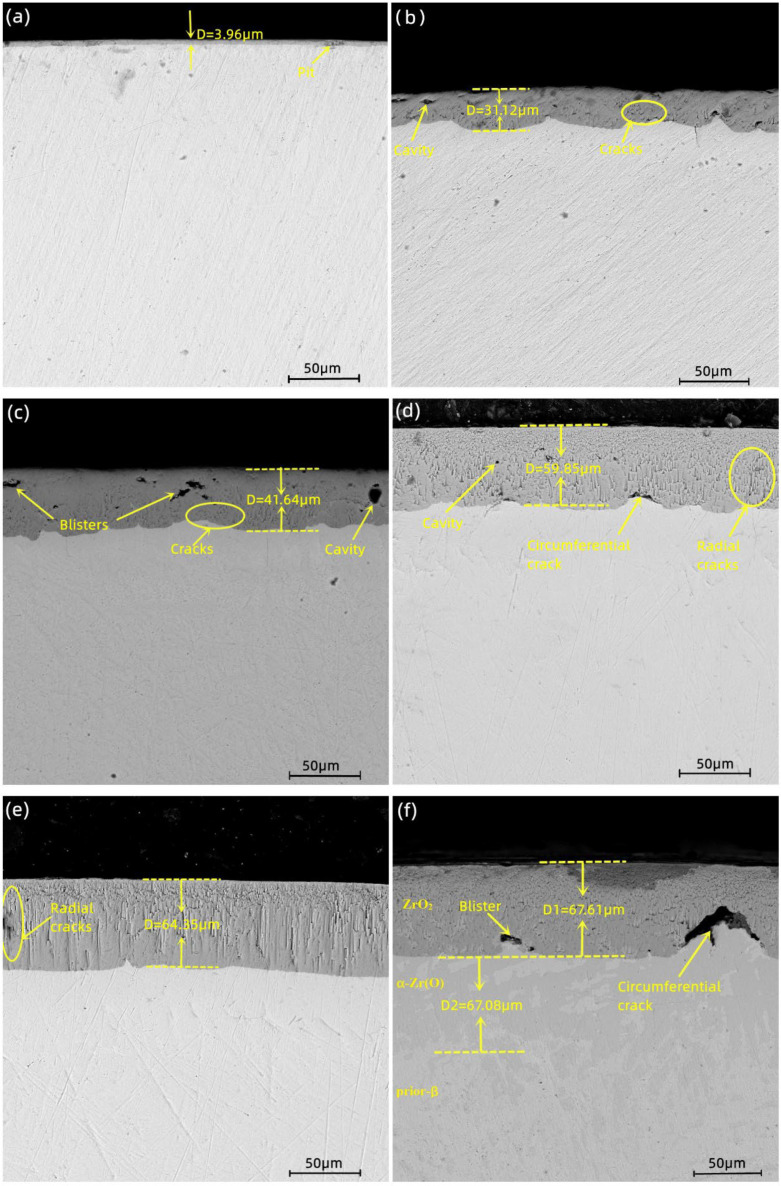
Sectional morphologies of Zr−Sn−Nb alloy after steam oxidation at 1050 °C for (**a**)100 s, (**b**) 500 s, (**c**) 1000 s, (**d**) 2500 s, (**e**) 4000 s, and (**f**) 5000 s.

**Figure 6:**
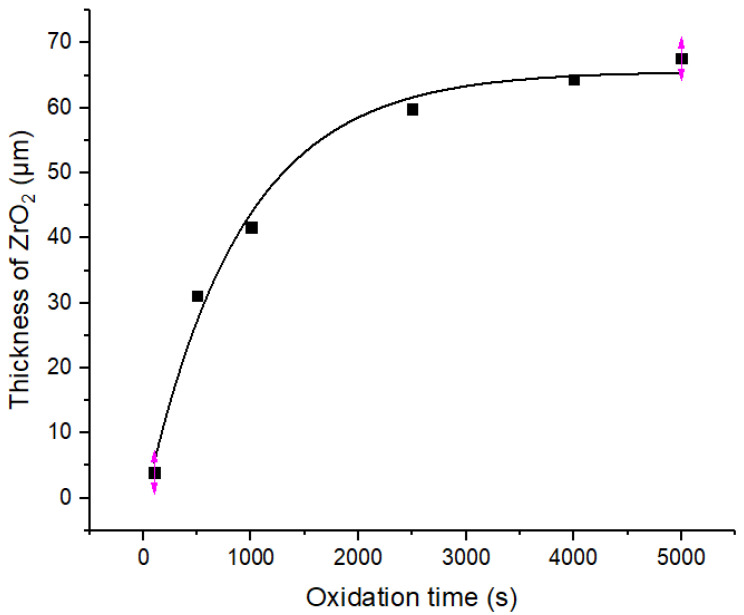
Variation curve of ZrO_2_ thickness of the Zr−Sn−Nb alloy with steam oxidation at 1050 °C.

**Figure 7:**
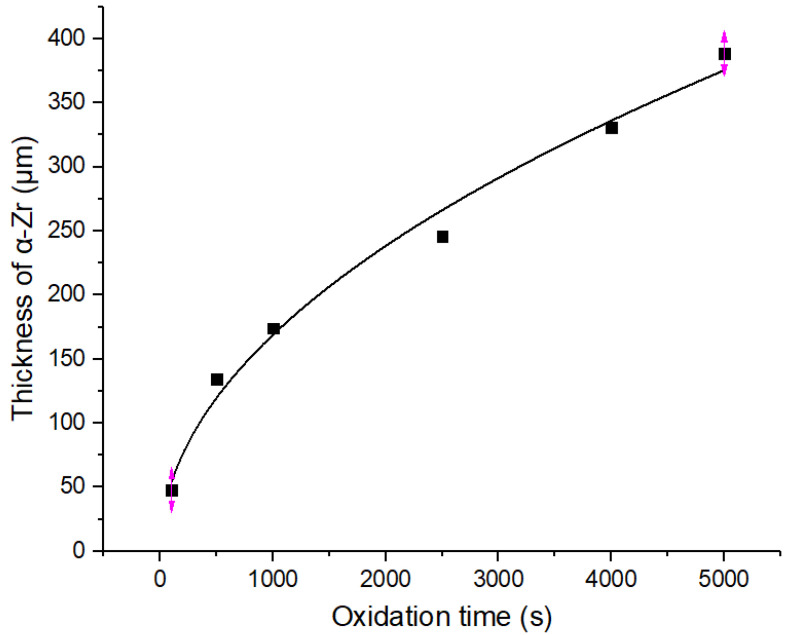
Variation curve of α-Zr thickness of the Zr−Sn−Nb alloy with steam oxidation at 1050 °C.

The cross-sectional morphology of the Zr−Sn−Nb alloy after steam oxidation at 1050 °C for 100 s, 500 s, 1000 s, 2500 s, 4000 s, and 5000 s is shown in Figure 5. According to the EDS results, the main microstructures of the Zr−Sn−Nb alloy cross-section included the brittle zirconium oxide (ZrO_2_) layer, the brittle and oxygen-rich α-Zr(O) metal phase, and the plastic prior-β metal phase. It can be seen that there was a clear boundary between the oxide layer (ZrO_2_), the α-Zr(O) phase, and the prior-β phase. In the initial stage, the boundary line type was relatively flat. The thickness of the oxide film increased with the increasing oxidation time. The thickness variation curve of the oxide layer is shown in Figure 6, and Figure 7 shows the thickness variation curve of α-Zr(O). We found that the curves of both the thickness of the oxide layer and the thickness of α-Zr(O) followed a parabolic law. As the oxide layer’s thickness increased, micropores, cracks, and grooves appeared in the oxide film. This indicates that the oxidation process was inhomogeneous, and the hydrogen produced during the reaction dissolved in the oxide film, expanding and breaking in volume, as described by Equation (3). Then, the prior-β in the cross-sectional organization of specimens after steam oxidation at 1050 °C was the only metallic phase to retain plasticity. However, the hydrogen produced by the reaction in the high-temperature steam environment increased the solid solution of oxygen in the β-phase and the rate of oxygen diffusion into this phase, eventually causing the embrittlement of the β-phase, which is reflected in the embrittlement fractures of macroscopic specimens.

(3) $$\mathrm{Zr}+2H_{2}O={\mathrm{ZrO}}_{2}+2H_{2}↑$$

Figure 5a,b show the cross-sectional structures of steam oxidation at 1050 °C for 100 s and 500 s. It can be seen that the oxide layer’s thickness was relatively low, as shown in Figure 5a. The growth interface of the α-Zr(O) and prior-β structures was smoother, and the prior-β phase region was thicker and denser to maintain a certain plasticity. In addition, the elongated needle-like martensite was present in the region of α-Zr(O) phase and the prior-β phase of both Figure 5a,b. With the increasing oxidation time (Figure 5c–f), the α-Zr(O) growth interface gradually became rough, and the α-Zr(O) phase (massive area) and the prior-β phase delaminated significantly (Figure 5f). In addition, this Zr−Sn−Nb-like zirconium alloy displayed two types of oxide film defects. One was the presence of nearly circular cavities in the oxide film, as shown in Figure 5c. The other was the presence of high-shape-ratio cracks in the oxide film, as shown in Figure 5d–f.

### 3.5. TEM Results of the Zr−Sn−Nb Alloy

**Figure 8:**
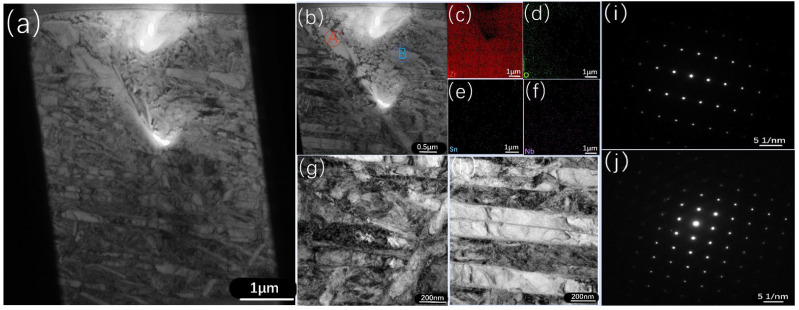
TEM results and EDS maps of the Zr−Sn−Nb sample oxidized at 1050 °C for 4000 s: (**a**,**b**) low-magnification TEM image of the middle area of the cross-section; (**c**–**f**) EDS maps of the area (**b**); (**g**,**h**) magnified views of areas in (**a**); (**i**) selected area electron diffraction (SAED) pattern of Point A in (**b**); (**j**) SAED pattern of Point B in (**b**).

Figure 8 shows the cross-sectional results of the Zr−Sn−Nb alloy, detected by TEM, when oxidized at 1050 °C for 4000 s. The cross-sectional samples were prepared by using the focused-ion-beam (FIB) method. From Figure 8c–f, it can be observed that the distribution of O elements was denser in the left half of the region than in the right half, and the diffusion direction was from left to right. The distribution of both Sn and Nb elements was relatively uniform. The organization of Figure 8g,h shows the slating, the high density of dislocations, and a small number of grains in an equiaxed shape. When the EDS and the electron diffraction results of Figure 8i,j are combined, it can be seen that that the second phase had a face-centered cubic structure of (Zr, Nb)_2_Fe and a dense hexagonal structure of Zr(Nb, Fe)_2_.

### 3.6. Cross-Sectional Distribution of Elements

**Figure 9:**
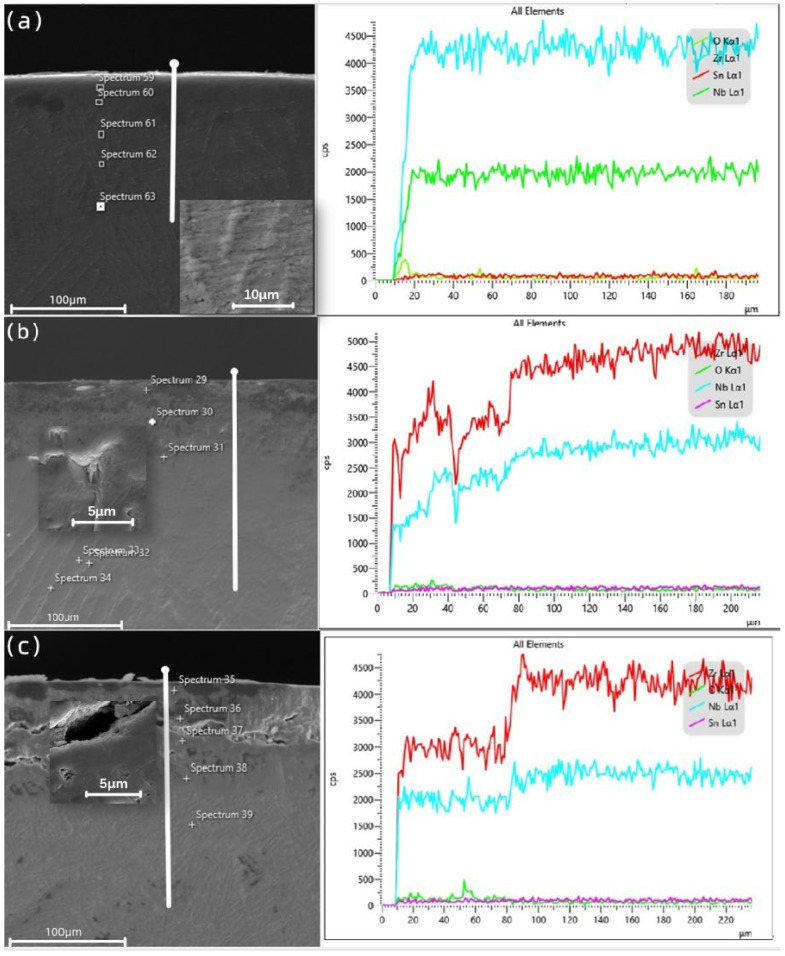
Cross-sectional point sweep region and BSE image of Zr−Sn−Nb alloy oxidized for (**a**) 100 s, (**b**) 2500 s, and (**c**) 5000 s. EDS line scanning at the position of the white line.

Figure 9a–c show the cross-sectional morphology and EDS line scanning results of the Zr−Sn−Nb samples at 100 s, 2500 s, and 5000 s of oxidation, respectively. The EDS point analysis results of P59~P63 are listed in Table 3. The EDS point analysis results of P29~P34 are listed in Table 4. The results of EDS point analysis for P35~P39 are listed in Table 5. Similarly to Figure 5f, all three samples show a three-layer structure. Based on the EDS line scan mapping (Figure 9a–c) and point analysis (Table 3, Table 4 and Table 5), as well as previous studies [30] by other researchers, we can confirm that the three layers, from the outside to the inside, were zirconia (ZrO_2_), α-Zr(O), and prior-β. In the early stage of high-temperature steam oxidation, the oxide film’s thickness on the cross section of the Zr−Sn−Nb alloy is low, and it is structurally intact. As shown in Table 3, the atomic contents of O, Zr, Nb, and Sn were mutated at the oxide film’s partition interface. However, the atomic number ratio of zirconium to oxygen is greater than 2, indicating that that a certain amount of Zr remained in the oxide film during the oxidation process. In Figure 9b, the oxide film thickness is significantly larger and the number of cracks has increased. From Table 4, it can be seen that the atomic proportions of O and Zr changed sharply compared to the oxidation time of 100 s, while the atomic proportions of Nb increased. Nb is a β-phase stable element. When the content of Nb increased, the oxidation resistance of the alloy decreased due to the influence of the second phase which formed.

After 5000 s of steam oxidation, the structure of the oxide layer was destroyed. At the same time, extensive cracks appeared and the oxide layer lost its ability to protect the internal structure. As can be seen in Table 5, the ratio of Zr to O atoms was 1, indicating that the large amount of oxygen solid solution increased the temperature required for the transition from α to β at high temperatures and long periods of steam oxidation, making the α-Zr(O) phase stable at high temperatures.

### 3.7. Surface XRD

**Figure 10:**
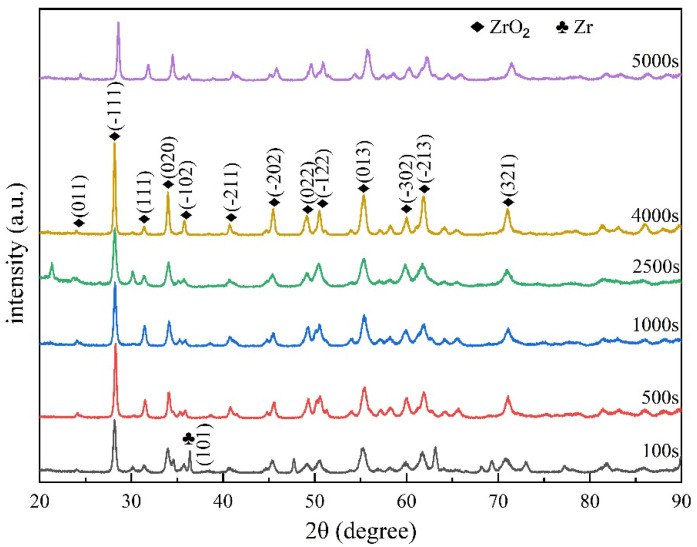
XRD patterns of the surface of the Zr−Sn-Nb alloy with oxidation for 100 s, 500 s, 1000 s, 2500 s, 4000 s, and 5000 s.

The surface phase compositions of the specimens after 100 s, 500 s, 1000 s, 2500 s, 4000 s, and 5000 s of oxidation were analyzed by X-ray diffraction (XRD), and the diffraction patterns are shown in Figure 10. It can be seen from the Figure that the oxides on the surfaces of the samples with different oxidation times after steam oxidation were mainly ZrO_2_, and there were only a few Zr residues on the surface of the sample with 100 s oxidation time. In addition, the specimens in this study were tubes, and a flatter area was taken from the surfaces of their walls for XRD inspection, so a partial peak phenomenon (peak of oxidation 5000 s in Figure 10) occurred. In conclusion, we learned that the surface Zr matrix oxidized more completely when the Zr−Sn−Nb alloy was oxidized by steam at 1050 °C.

## 4. Discussion

We studied the Zr−Sn−Nb cladding tubes using single-sided oxidation treatment. The increase in weight before and after oxidation; the structural integrity of the zirconia layer at different oxidation times; the change pattern of the thickness of the α-Zr(O) phase and the prior-β phase; and the main elements, namely, O, Zr, Nb, and Sn, in the cross section all had effects on the high-temperature steam oxidation properties of the Zr−Sn−Nb alloy.

As in other related studies, in the case of steam oxidation at 1050 °C, the cross-sectional organization at the beginning of oxidation was clearly layered and the structure of the oxide layer was relatively intact. As the oxidation time increased, the crystal structure of zirconia underwent a transformation at high temperatures. It was shown that the phase transformation was often accompanied by certain volume changes, which affected the changes in the internal stresses of the oxide layer, generating cracks and eventually affecting the structural integrity of the oxide layer, leading to the failure of the parabolic law followed by its oxidation kinetic curve. According to its macroscopic characteristics, the Zr−Sn−Nb alloy can ensure the integrity of the overall structure for a longer period of time during steam oxidation treatment at 1050 °C.

In addition, related studies have shown that Sn is an α-Zr(O) phase stable element which is mainly present in the zirconium alloy matrix as a solid solution atom. The appropriate amount of Sn can reduce the additional vacancy mobility generated by the replacement of oxygen ions by impure N in zirconium, and can enhance the oxidation resistance of the alloy. The optimized composition of many zirconium alloys is to reduce the Sn content on the basis of the original, as is the case for improved Zr-4 and optimized ZIRLO, but the Sn content should not be too low. In addition, Nb is a β-phase stable element with an extremely low solid solution in α-Zr(O). Relevant studies have shown that when the Nb content is closest to the equilibrium solid solution degree of the alloy, and the Sn content is also moderate, the oxidation resistance of Zr−Sn−Nb is stronger than that of other conventional zirconium alloys.

## 5. Conclusions

In this paper, the isothermal oxidation behavior of Zr−Sn−Nb alloys under a steam environment at 1050 °C was investigated. The macroscopic appearance, weight gains, microscopic surface, and cross-sectional morphologies of the samples after different oxidation times were analyzed. Based on the experimental results, the main conclusions of this study are as follows:

(1) During high-temperature steam oxidation at 1050 °C, the surface of the Zr−Sn−Nb alloy undergoes a color change and local deformation, but is able to maintain an intact macrostructure for a long time until it breaks down after 4000 s of oxidation.
(2) When the Zr−Sn−Nb alloy is oxidized by steam at 1050 °C, the curve of oxidation weight gain versus time follows a parabolic law. In addition, the curves of ZrO_2_ thickness and α-Zr thickness also follow a parabolic law.
(3) The microstructure of the Zr−Sn−Nb alloy after steam oxidation at 1050 °C consists mainly of the oxide layer (ZrO_2_), α-Zr(O), and prior-β. The breakdown of oxide films, the generation of micropores and cracks, and the increase in the number of cracks are related to the increase in the proportions of ZrO_2_ and α-Zr(O).
(4) The changes in the atomic content of O, Zr, Nb, and Sn at all sections of the alloy’s cross-section become more dramatic with increasing oxidation time. As the oxidation time increases, the proportion of Nb atoms becomes greater, leading to a decrease in the oxidation resistance of the alloy.

## Figures and Tables

**Table 1:** Oxidation weight gain of Zr−Sn−Nb alloy at 1050 °C for different times.

Oxidation Time (s)	100	500	1000	2500	4000	5000
Weight gain (mg/cm^2^)	0.20	0.65	0.98	1.40	2.10	2.95

**Table 2:** Function of oxidation weight gain curve of Zr−Sn−Nb alloy.

Oxidation Temperature (°C)	1050 °C
Oxidation time (s)	0 s~5000 s
Equation	$W=at^{b}$
a	0.0057
b	0.7239
R-Squared	0.9612

**Table 3:** EDS point analysis at the marked positions in Figure 8a.

Position	Element Content	(at%)		
	O	Zr	Nb	Sn
Spectrum 59	28.00	69.07	2.41	0.52
Spectrum 60	19.72	76.74	3.25	0.29
Spectrum 61	20.40	75.97	3.16	0.47
Spectrum 62	24.03	72.73	2.82	0.42
Spectrum 63	17.65	78.60	3.22	0.53

**Table 4:** EDS point analysis at the marked positions in Figure 8b.

Position	Element Content	(at%)		
	O	Zr	Nb	Sn
Spectrum 29	45.69	50.70	3.20	0.42
Spectrum 30	26.94	59.15	13.53	0.38
Spectrum 31	15.73	73.04	10.68	0.55
Spectrum 32	13.26	73.81	12.59	0.33
Spectrum 33	11.30	77.42	11.08	0.19
Spectrum 34	19.01	68.32	12.24	0.44

**Table 5:** EDS point analysis at the marked positions in Figure 8c.

Position	Element Content	(at%)		
	O	Zr	Nb	Sn
Spectrum 35	43.82	47.18	8.54	0.46
Spectrum 36	43.19	48.04	8.27	0.51
Spectrum 37	38.57	51.97	9.11	0.34
Spectrum 38	16.45	73.43	9.53	0.60
Spectrum 39	11.66	77.41	10.11	0.81

## Data Availability

The raw/processed data required to reproduce these findings cannot be shared at this time, as the data also forms part of an ongoing study.
